# Supplementary material for: Human navigation strategies and their errors result from dynamic interactions of spatial uncertainties
Source: Nat Commun. 2024 Jul 6;15:5677. doi: 10.1038/s41467-024-49722-y (PMC11227593; doi:10.1038/s41467-024-49722-y)
Supplement: Supplementary file 1 — Supplementary Information [file 41467_2024_49722_MOESM1_ESM.pdf]

Supplementary Information

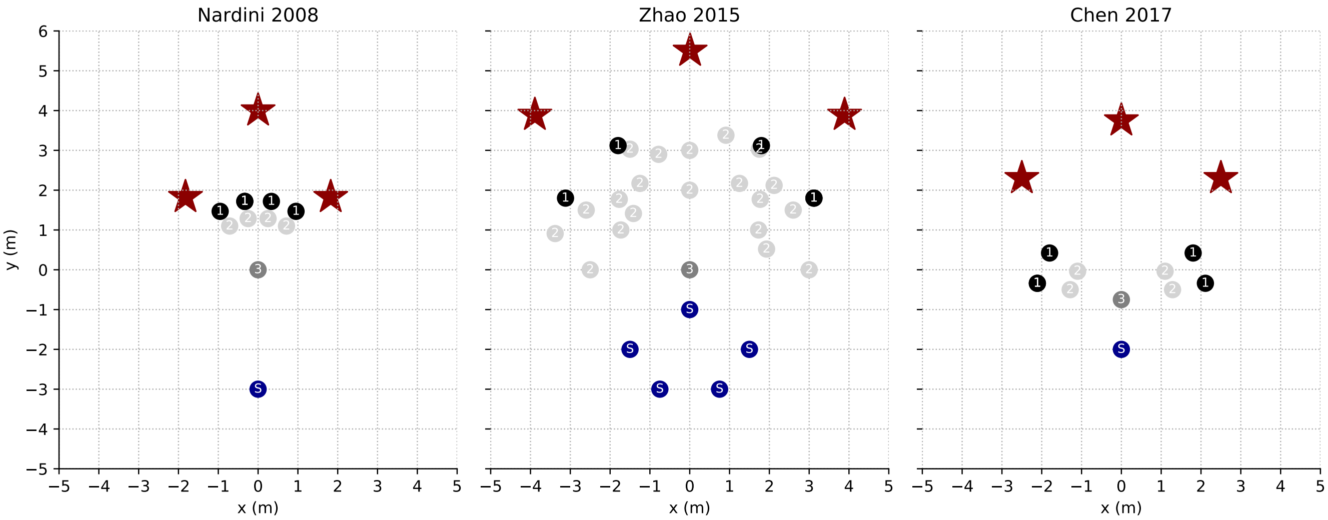

Supplementary Figure 1. Environmental layouts in [1–3] with location of starting position, goal posts and landmarks. Different combination of goal locations lead to different three legged outbound paths. Blue dots indicate possible start locations. Black dots indicate possible homing locations. Grey dots indicate possible second target locations. Darkgrey dots indicate the location of the last target. Stars indicate position of landmark. For the distal landmark conditions in Zhao et al. 2015 [2] landmark locations were moved 500m, while maintaining the same angular configuration. For the one landmark environment in Chen et al. 2017 [3] only the middle landmark was shown during outbound and homing paths respectively.

Study / Parameter	Nardini et al. 2008 [1]	Chen et al. 2017 [3]	Zhao et al. 2015 [2]
Environment size	6 x 10 m	6.9 x 8.8m	10 x 10m
Viewing modality	Regular vision	HMD / VR	HMD / VR
Field of view	180°	60°	63°
Number of landmarks	3	1 or 3 (poor vs. rich)	3 (proximal vs. distal)
Target visibility at start	all at once	sequentially	sequentially
Target jitter	none	plus or minus 0.4m	none
Homing distance	1.75m	2.15m	3.6m
Number of trials per condition (per participant)	4 (16 total)	10 (40 total)	40 (360 total)
Waiting time (all conditions)	8 seconds	20 seconds	10 seconds
Landmark rotation (conflict)	15° (left and right)	15° (left and right)	15°, 30°, 45°, 90° (left and right)
Disorientation turning speed (landmark)	$\sim 90^{\circ}/s$	$\sim 90^{\circ}/s$	$\sim 73^{\circ}/s$
Disorientation position (landmark)	0.5m	extracted from trajectory data	extracted from trajectory data
Reorientation prior homing (landmark)	yes	no	no

Supplementary Table 1. Experimental task parameters. Task parameters were extracted from the methods section of the respective paper, the trajectory data (if available), or further clarified in correspondence with the respective studies original authors.

Parameter	Notation	Value	Description	Reference
**Motor noise**	$\boldsymbol{\alpha}$			
motor_linear_linear	α_1	0.1	linear-linear velocity noise (stepping)	[4]
motor_linear_angular	α_2	0.001	linear-angular velocity noise (veering)	[4, 5]
motor_angular_linear	α_3	0.02	angular-linear velocity noise (translating)	
motor_angular_angular	α_4	0.1	angular-angular velocity noise (rotating)	[6]
**Observation noise**	$\boldsymbol{\epsilon}$			
obs_distance_min	$\sigma_{r_{min}}$	0.0225	minimum distance noise (central)	[7, 8]
obs_distance_max	$\sigma_{r_{max}}$	0.1875	maximum distance noise (periphery)	[7, 8]
obs_bearing_min	$\sigma_{\psi_{min}}$	0.045	minimum bearing noise (central)	[7, 8]
obs_bearing_max	$\sigma_{\psi_{max}}$	0.225	maximum bearing noise (periphery)	[7, 8]
**Representation noise**				
representation	δ_{xy}	0.01295^2	representation noise	
**Cost-Function weights**	**w**			
	w_1	7.5	state cost J_{goal}	
	w_2	0.25	position uncertainty cost $J_{position-uncertainty}$	
	w_3	0.25	map uncertainty cost $J_{map-uncertainty}$	
	w_4	2.5	control cost J_{goal}	
	$\mathbf{C_q}$	diag([1.0, 1.0, 0.0])	individual state cost (x,y,heading)	
	$\mathbf{C_r}$	diag([1.0, 0.0])	individual control cost (translation; rotation)	
	C_{tr}	0.25	individual control cost (translation & rotation)	
**Motion limits**				[2, 3, 9]
	v_{max}	1.22	maximum linear velocity (m/s)	
	v_{min}	0.0	minimum linear velocity (m/s)	
	w_{max}	270.0	maximum angular velocity (deg/s)	
	w_{min}	-270.0	minimum angular velocity (deg/s)	
	dv_{max}	0.25	maximum linear acceleration (Δ m/s)	
	dw_{max}	$\frac{\pi}{4}$	minimum angular acceleration (Δ deg/s)	
**Initial belief state**				
	$\boldsymbol{\mu}_{\text{pose}}$	$N \sim (x_0, \text{diag}([0.5^2, 0.5^2, 0.05^2]))$	initial position belief (x,y,theta)	
	$\boldsymbol{\mu}_{\text{map}}$	$x_{0\,\text{map}}$	initial map belief (re-initialized with observations)	
**Initial uncertainty**				
	$\boldsymbol{\Sigma}_{\text{pose}}$	diag($[0.5^2, 0.5^2, 0.05^2]$)	initial positional uncertainty (m)	
	$\boldsymbol{\Sigma}_{\text{map}}$	diag([1000, 1000] * n_landmarks)	initial landmark uncertainty (m)	
**Algorithm parameters**				
	T	15	planning horizon (n timesteps)	
	P	5	control horizon (n timesteps)	

Supplementary Table 2. Model parameters for the dynamic Bayesian actor model.

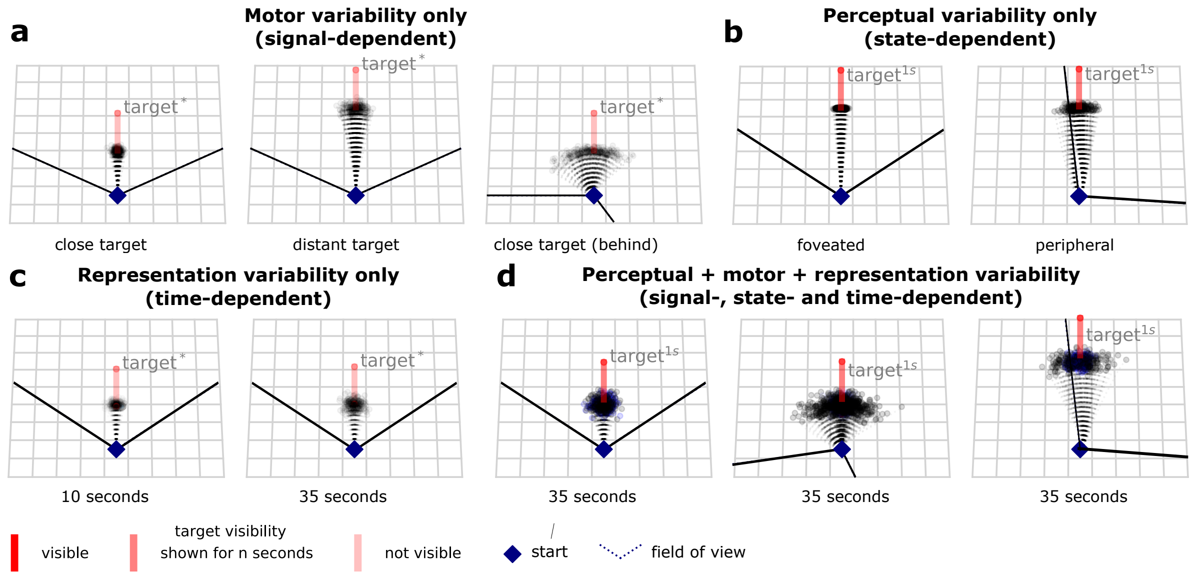

Supplementary Figure 2. Simulating sources of variability in goal-directed navigation behavior. a Effect of signal-dependent motor noise when walking to a previously seen target. Different distances (left, middle) or initial headings (right) lead to different endpoint variability due to noise in linear and angular velocities. **b** State-dependent noisy observations of the same target (visible for 1s) viewed centrally (left) or viewed in the periphery of the visual field (right). **c** Time-dependent noise accrual in internal representation and subsequent movement to the target shown for 10s (left) and 35s (right). **d** Combined effect of noise in action, perception, and representation on endpoint variability. Participants either face the target directly (left), the target is behind them and they store a representation of its location (middle) in memory, or they see the target only in the noisy periphery (right).

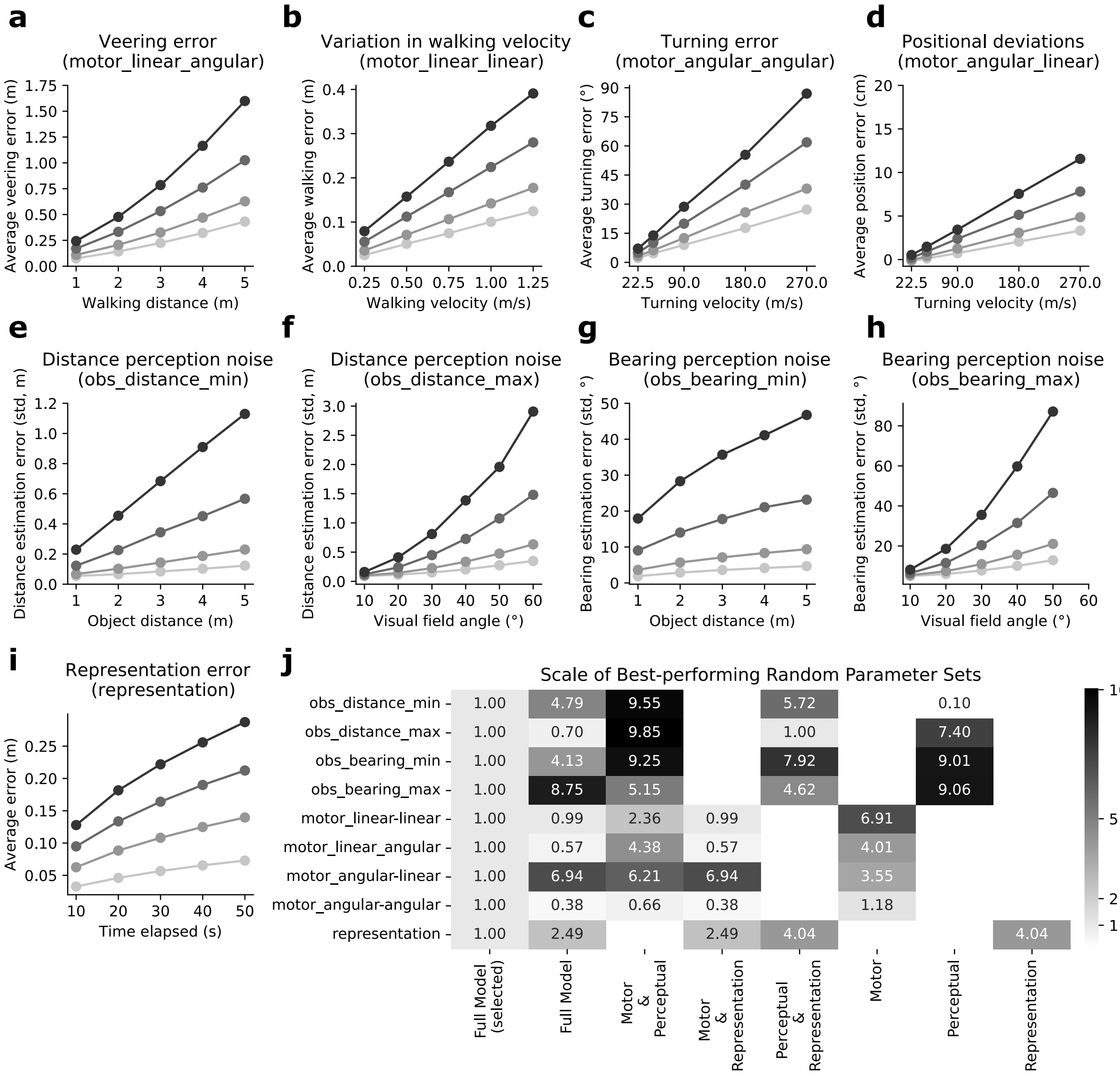

	Full Model (selected)	Full Model	Motor & Perceptual	Motor & Representation	Perceptual & Representation	Motor	Perceptual	Representation
obs_distance_min	1.00	4.79	9.55		5.72		0.10	
obs_distance_max	1.00	0.70	9.85		1.00		7.40	
obs_bearing_min	1.00	4.13	9.25		7.92		9.01	
obs_bearing_max	1.00	8.75	5.15		4.62		9.06	
motor_linear-linear	1.00	0.99	2.36	0.99		6.91		
motor_linear_angular	1.00	0.57	4.38	0.57		4.01		
motor_angular-linear	1.00	6.94	6.21	6.94		3.55		
motor_angular-angular	1.00	0.38	0.66	0.38		1.18		
representation	1.00	2.49		2.49	4.04			4.04

Supplementary Figure 3. Effect of parameter scales (1x, 2x, 5x, 10x) on motor, perceptual and representational variability a Lateral deviation from walking straight with increasing walking distance for different scales of α_2. **b** Variability in walking speed for a 1s walking bout as a function of walking speed for different scales of α_1. **c** Average turning error for a 1-second turn at varying turning speeds at different parameter scales of α_3 **d** Lateral deviation in position when turning on the spot for a 1-second turn for different scales of α_4. **e** Average distance perception error for a centrally viewed object with increasing object distance for different parameter scales of $\sigma_{r_{min}}$ **f** Average distance perception error for a peripherally viewed object (3m distance) with increasing visual field angle $\sigma_{r_{max}}$ at different parameter scales. **g** Average bearing perception error for increasing object distance for different parameter scales of $\sigma_{\psi_{min}}$. **h** Average bearing perception error for peripherally viewed object (5m distance) with increasing visual field angle for different parameter scales of $\sigma_{\psi_{max}}$. **i** Representation error as a function of time for different parameter scales of δ_{xy}. **j** Parameter values of best-performing random parameter sets across different models (50 per model) for the experiment of Nardini et al. 2008.

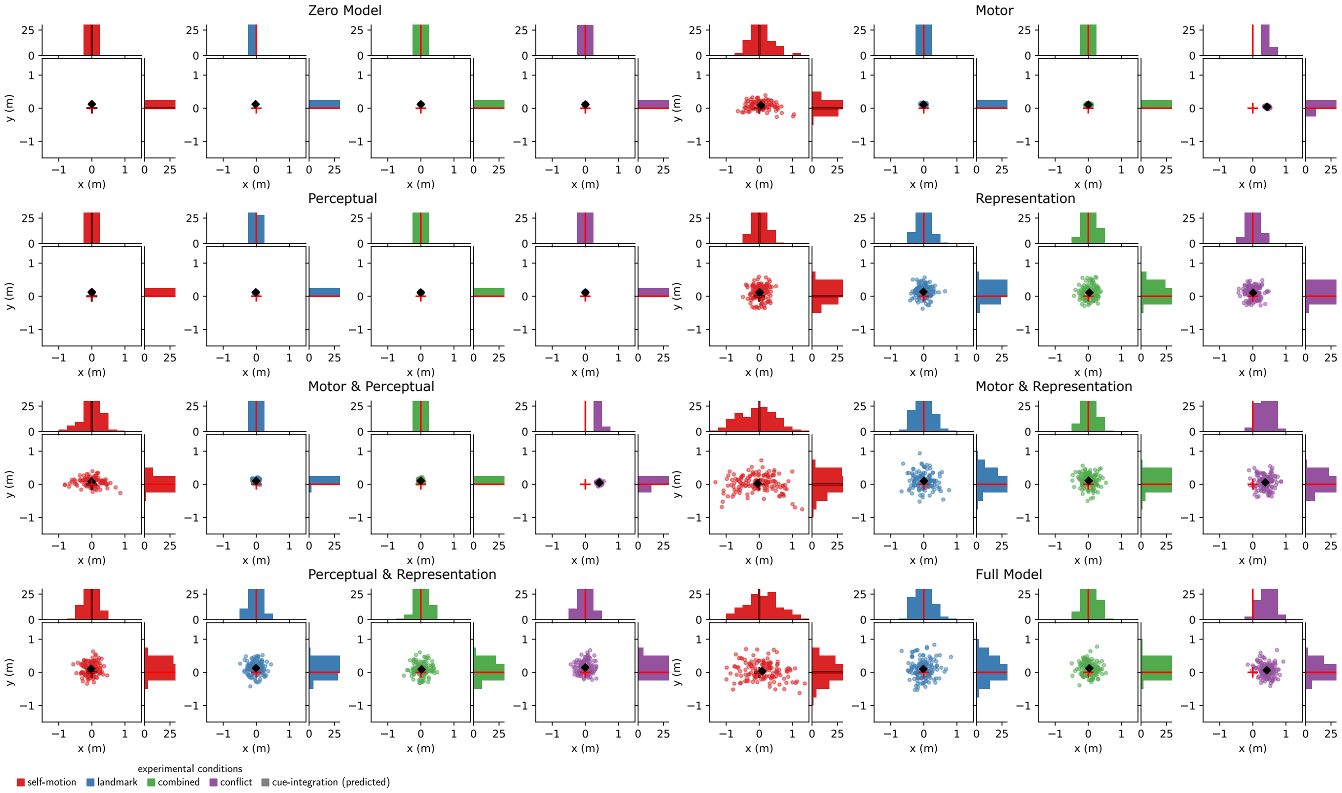

Supplementary Figure 4. Nardini et al. 2008 model ablation study. Endpoint distributions across cue conditions.

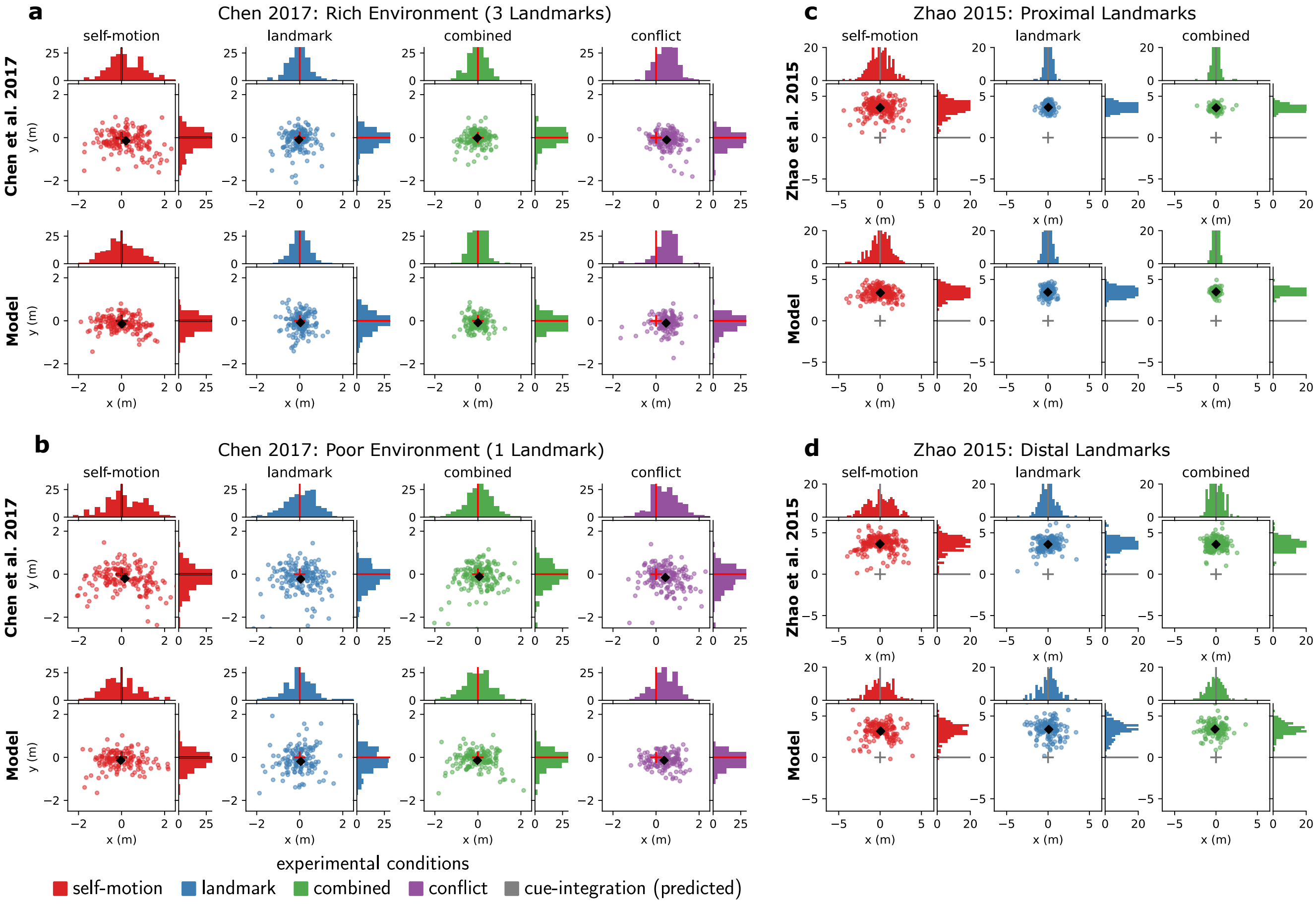

Supplementary Figure 5. Empirical vs. simulated endpoint variability for homing responses in Chen et al. 2017 and Zhao et al. 2015 (a and b) Endpoint distributions from Chen et al. 2017 [3] (top; a: Exp1a rich environment; b: Exp1a poor environment) and model simulations (bottom; a: Exp1a rich environment; b: Exp1a poor environment). Endpoint distributions show no statistically significant difference between our model and the empirical data for the environment with three landmarks (homogeneity energy test between conditions: self-motion ($p = 0.24$), landmark ($p = 0.627$), combined ($p = 0.136$), conflict ($p = 1.0$) and the environment with one landmark (homogeneity energy test between conditions: self-motion: ($p = 0.108$), landmark ($p = 1.0$), combined ($p = 0.535$), conflict ($p = 0.595$). **(c and d)** Endpoint Distribution from Zhao et al. 2015 [2] (top; c: proximal landmarks; d: distal landmarks) and simulated participants (bottom; c: proximal landmarks; d: distal landmarks). Comparison of endpoint distributions reveal statistically significant differences between our model and the empirical data for the proximal environment (homogeneity energy test between conditions: self-motion ($p = 0.012$), landmark ($p = 0.004$), combined ($p = 0.004$)) and the distal environment (homogeneity energy test between conditions: self-motion ($p = 0.004$), landmark ($p = 0.0008$), combined ($p = 0.044$)). Data for conflict condition are shown in Supplementary Figure 14.

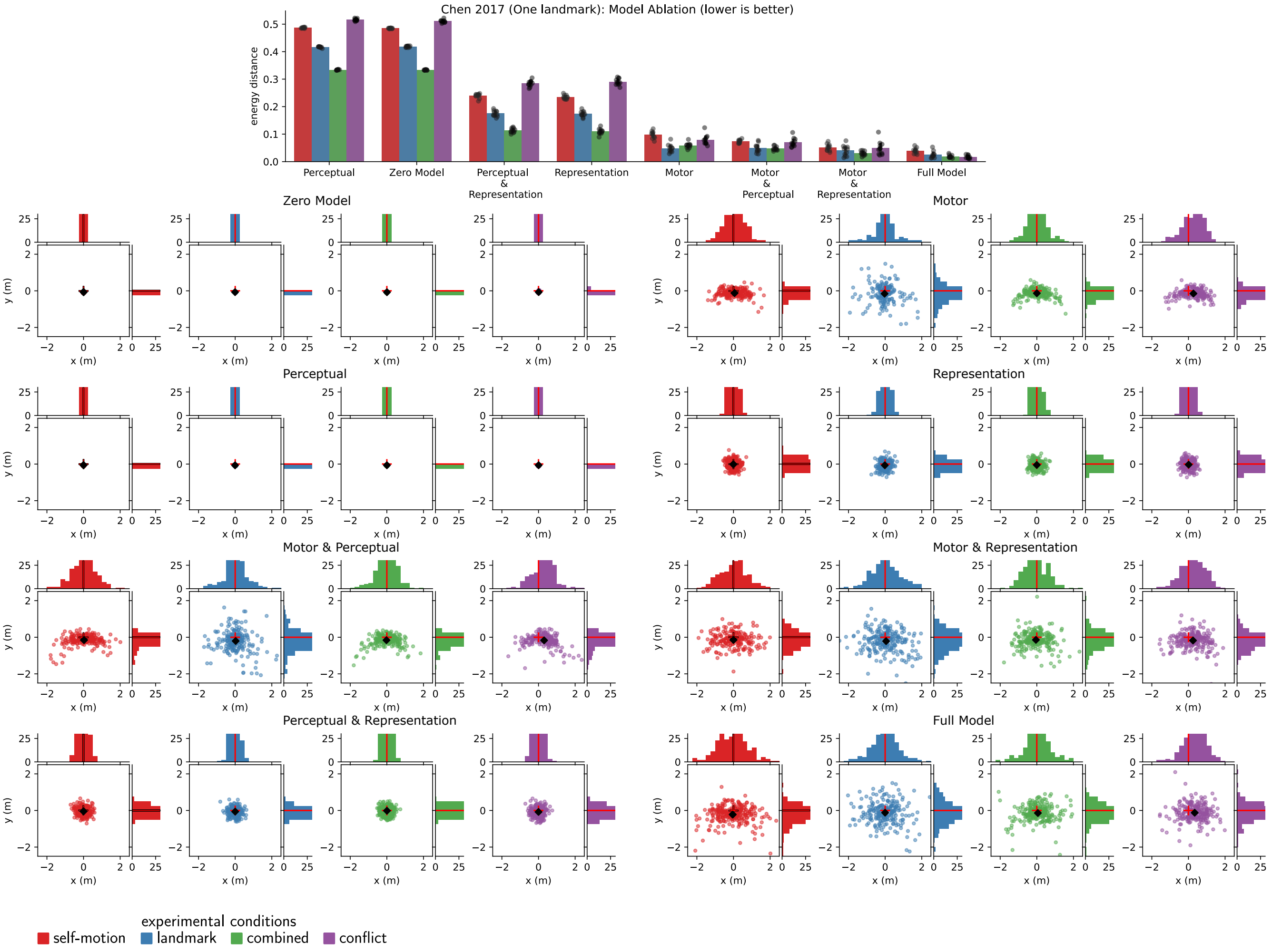

Supplementary Figure 6. Chen 2017 et al. model ablation study. Endpoint distributions for the one landmark environment (Chen et al. 2017). Energy distances were calculated between empirical and simulated data (n = 10) for each condition, with error bars indicating mean energy distance ± 1 SD. Source data are provided as a Source Data file.

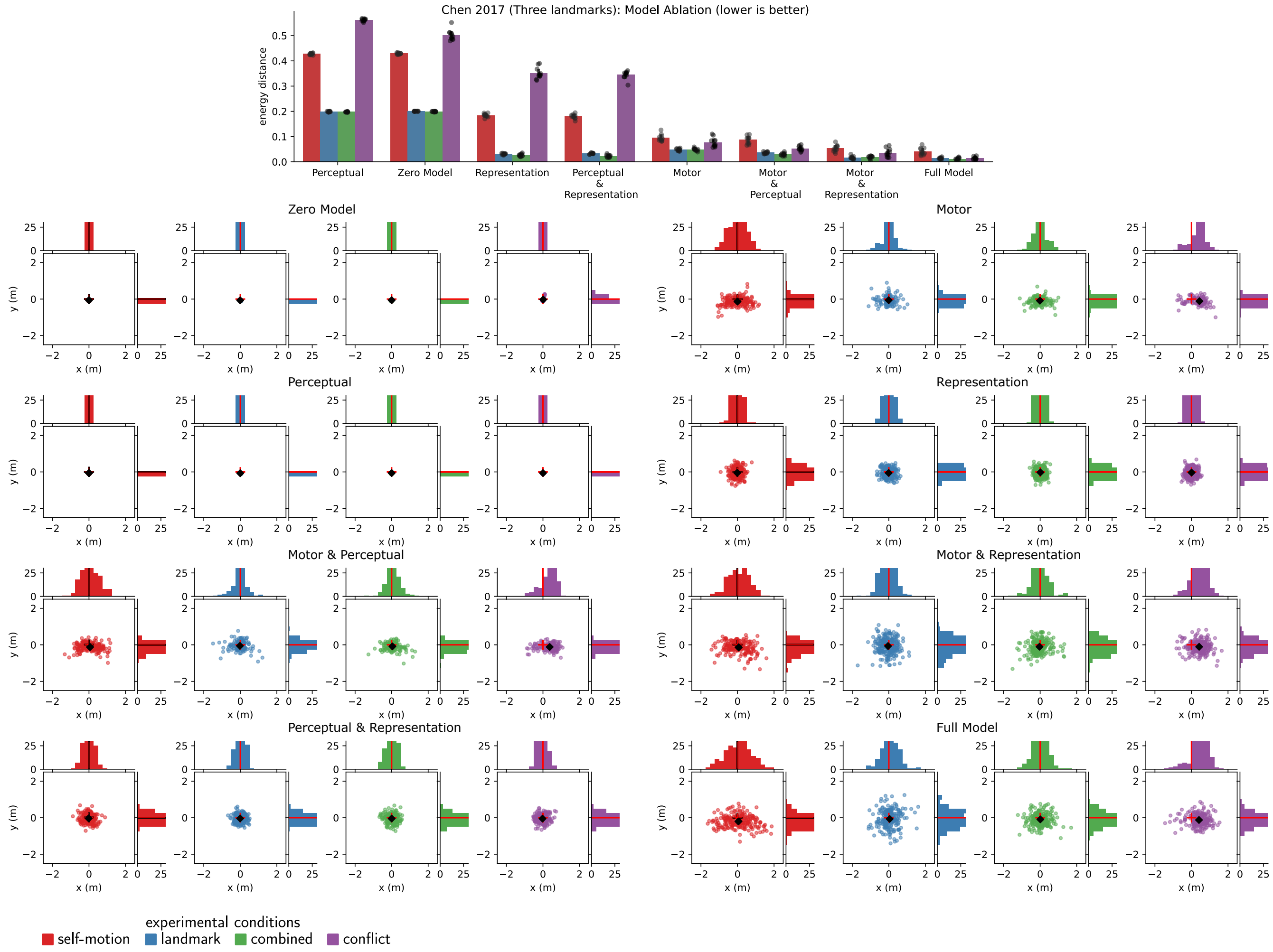

Supplementary Figure 7. Chen 2017 et al. model ablation study. Endpoint distributions for the three landmark environment (Chen et al. 2017). Energy distances were calculated between empirical and simulated data (n = 10) for each condition, with error bars indicating mean energy distance ± 1 SD. Source data are provided as a Source Data file.

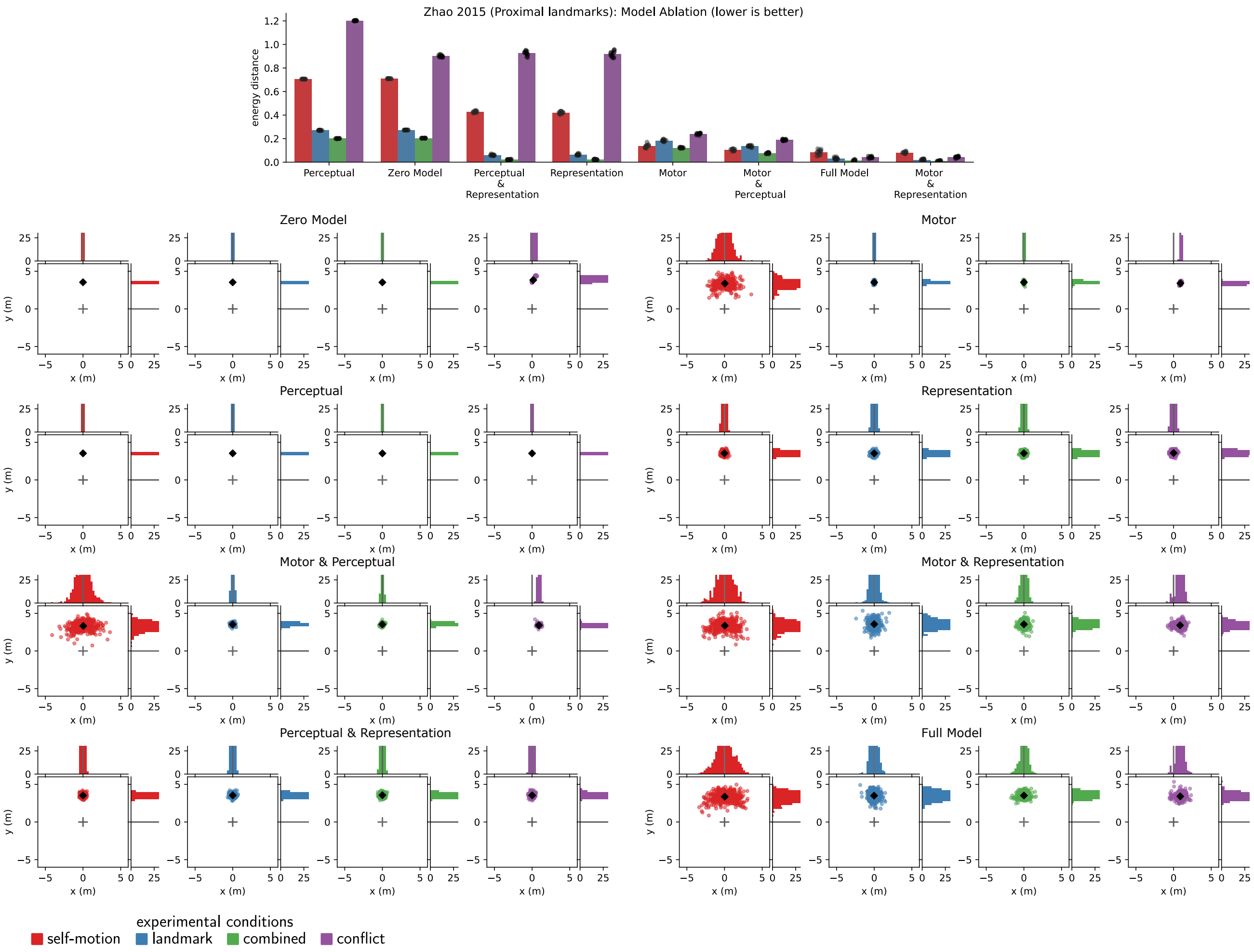

Supplementary Figure 8. Zhao et al. 2015 model ablation study. Endpoint distributions proximal environment (Zhao et al. 2015). Energy distances were calculated between empirical and simulated data (n = 10) for each condition, with error bars indicating mean energy distance ± 1 SD. Source data are provided as a Source Data file.

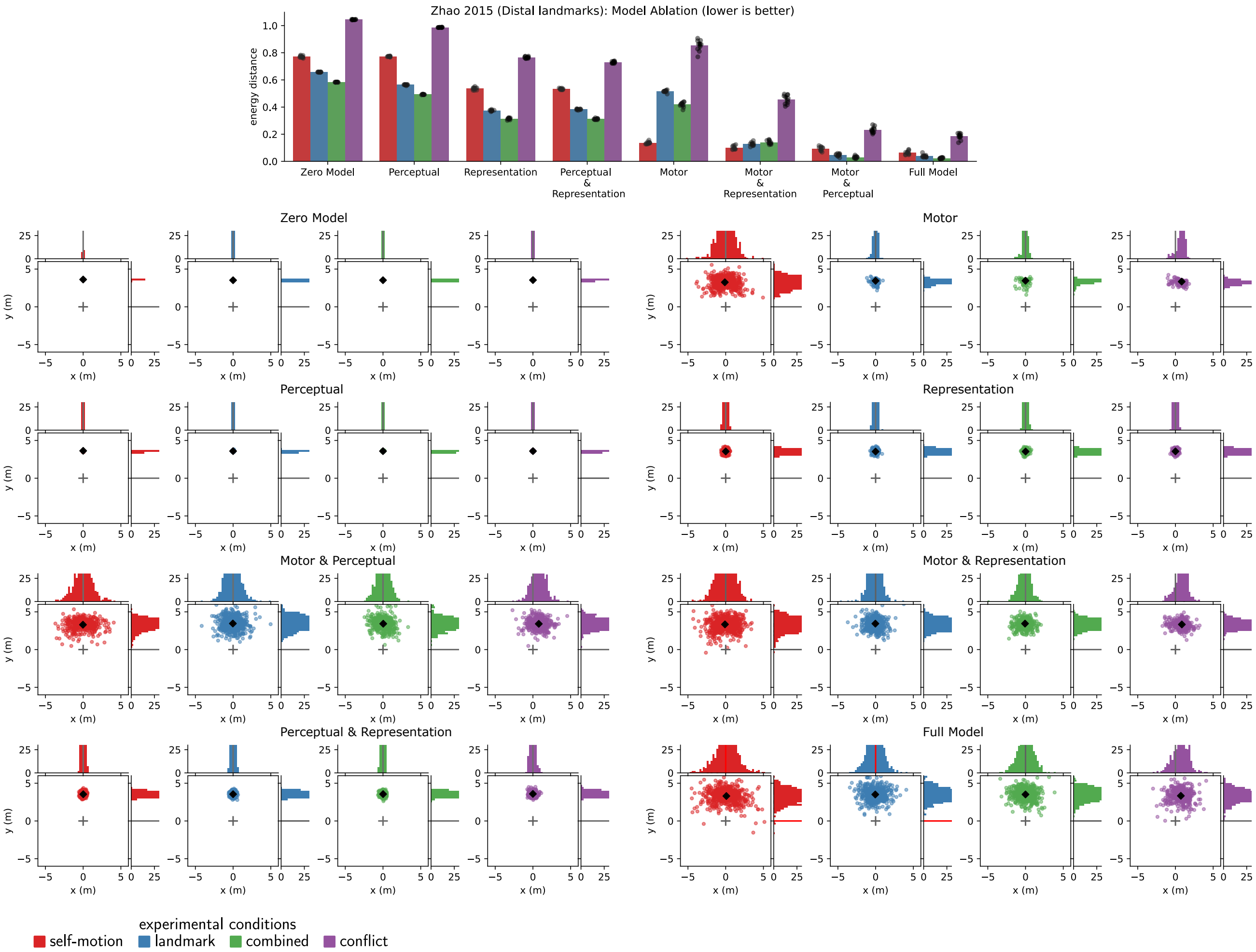

Supplementary Figure 9. Zhao et al. 2015 model ablation study. Endpoint distributions distal environment (Zhao et al. 2015). Energy distances were calculated between empirical and simulated data (n = 10) for each condition, with error bars indicating mean energy distance ± 1 SD. Source data are provided as a Source Data file.

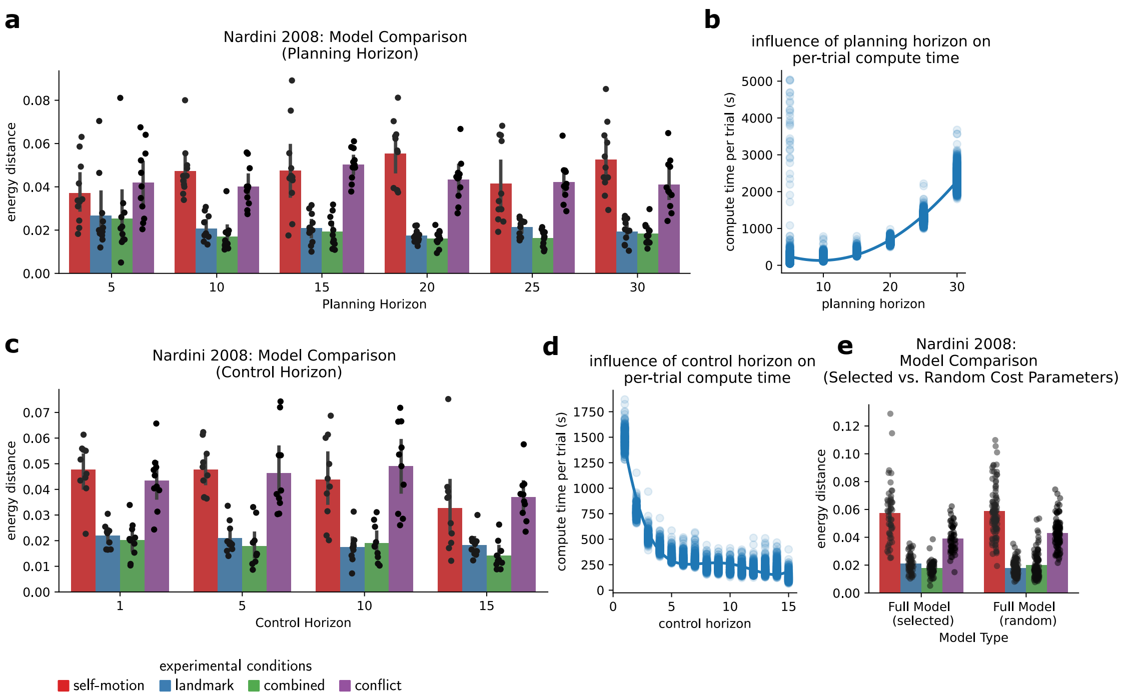

Supplementary Figure 10. Model comparison (planning and control horizons & cost-function parameters) a Comparison of the influence of different planning horizons (0.5, 1.0, 1.5, 2.0, 2.5, 3.0 seconds) on endpoint distributions for the homing task of Nardini et al. 2008. The control horizon was fixed at 5, and dt was set to 0.1 seconds, i.e., 0.5 seconds. **b** Per-trial compute times for different planning horizons. **c** Comparison of the influence of different control horizons (0.1, 0.5, 1 , 1.5 seconds) on endpoint distributions for the homing task of Nardini et al. 2008. Control horizon was set fixed at 15 and dt was set to 0.1 seconds, i.e., 1.5 seconds. **d** Per-trial compute times for different control horizons. **e** Random model comparison of different cost-function weights for Nardini et al. 2008. Source data are provided as a Source Data file.

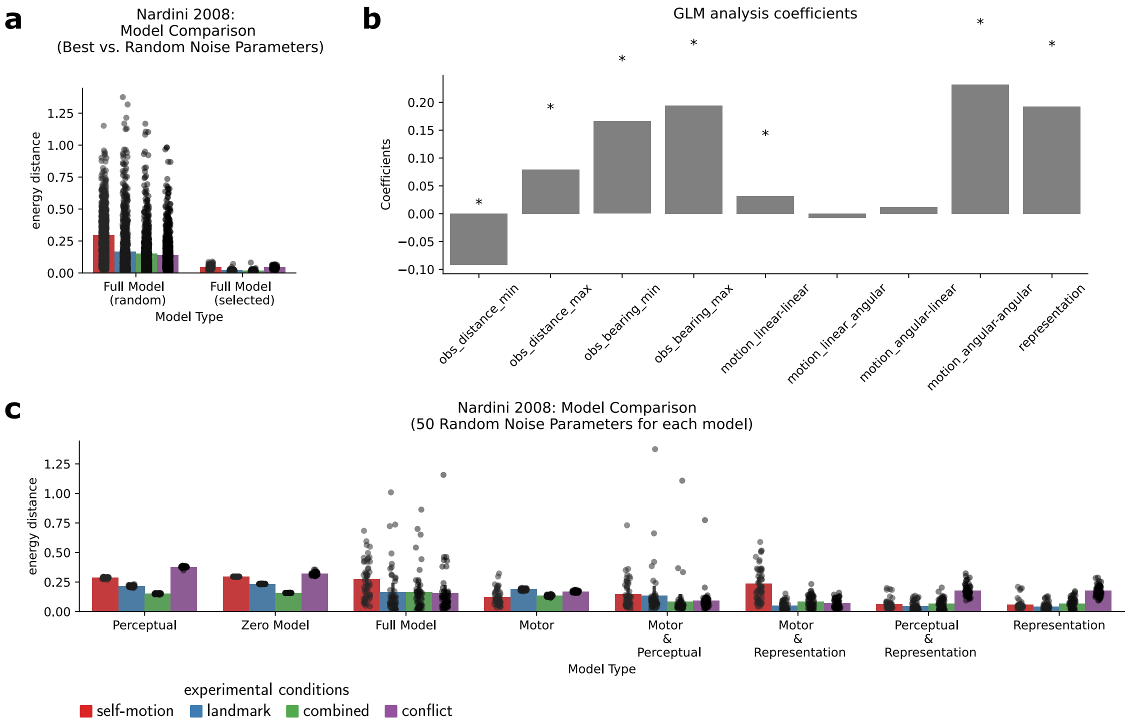

Supplementary Figure 11. Model comparison (random parameter sets) a Comparison of 1000 randomly sampled parameter sets versus selected model parameters for the homing task of Nardini et al. 2008. Random parameter sets were uniformly sampled between 0 and up to one magnitude higher of the selected parameter set. **b** GLM Analysis describing the impact of different parameters on the models ability to explain endpoint distribution for the homing task of Nardini et al. 2008. Asterisks denote significant correlations ($p < 0.05$). **c** Random parameter set model comparison with models containing different sources of variability and different numbers of noise parameters. Source data are provided as a Source Data file.

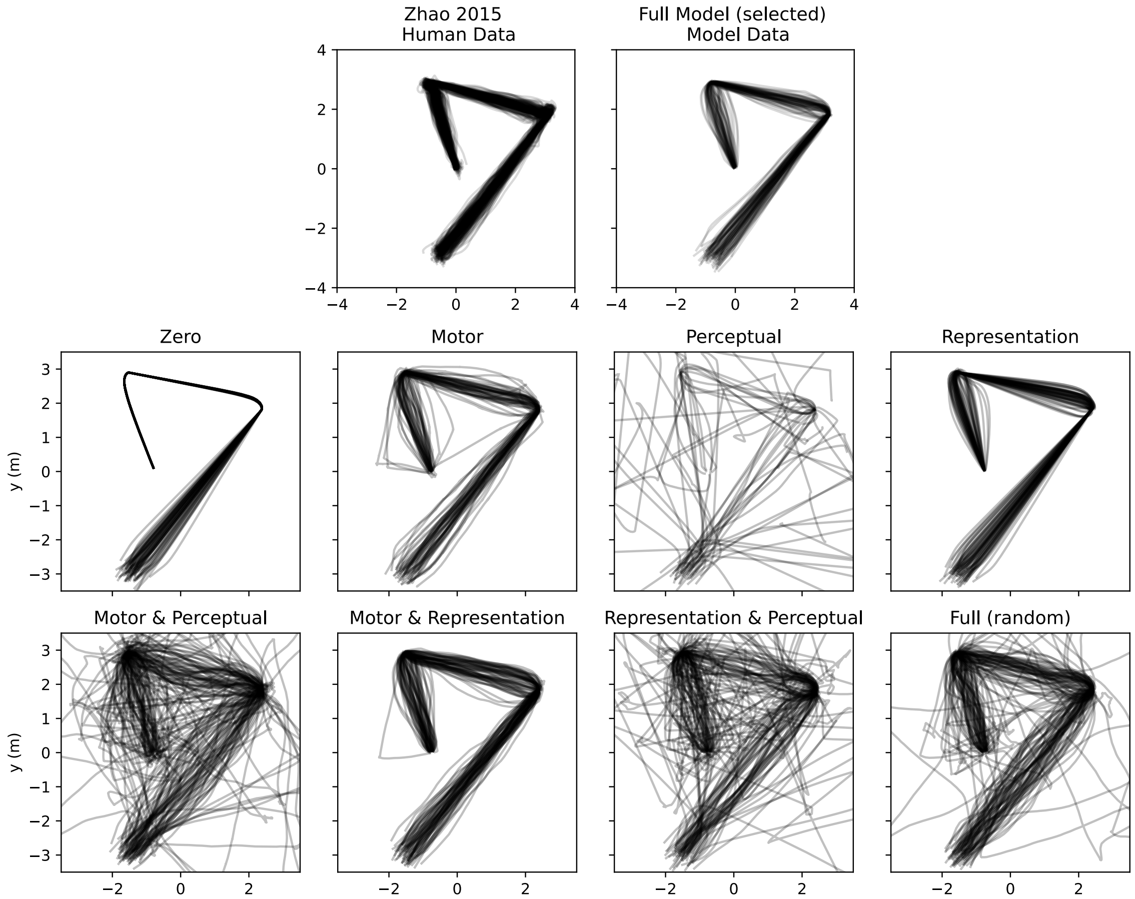

Supplementary Figure 12. Trajectories for Zhao et al 2015 proximal experiment: Human vs. model data. Trajectories were simulated for best-performing random parameter set for each of the eight different models.

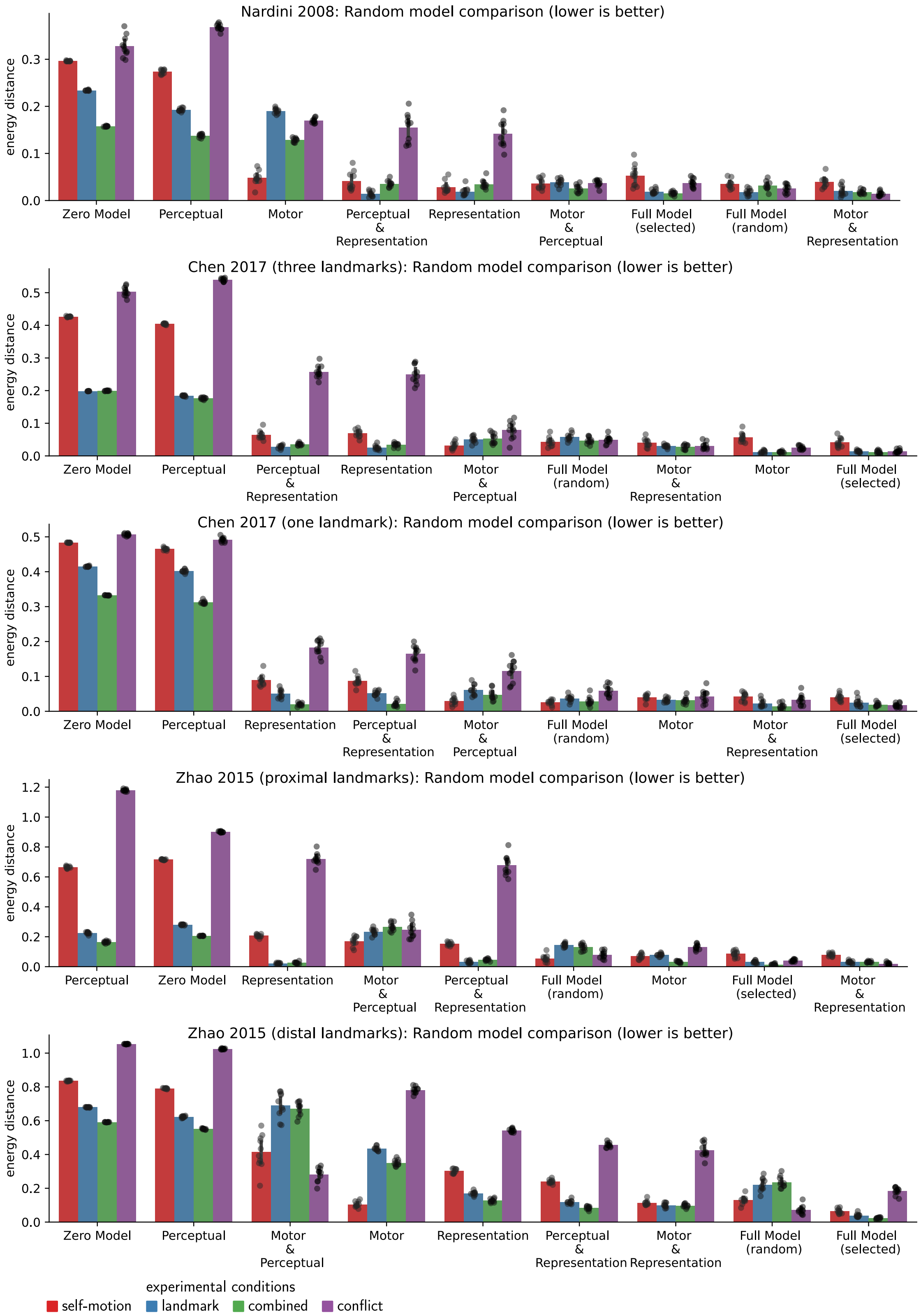

Supplementary Figure 13. Random model comparison across all experiments. For each of the eight different models the best-performing parameter sets in the task of Nardini et al. 2008 (Supplementary Figure 9c) were selected and subjected to transfer testing across experiments from Zhao et al. 2015 and Chen et al. 2017 (10 simulations for each model). Source data are provided as a Source Data file.

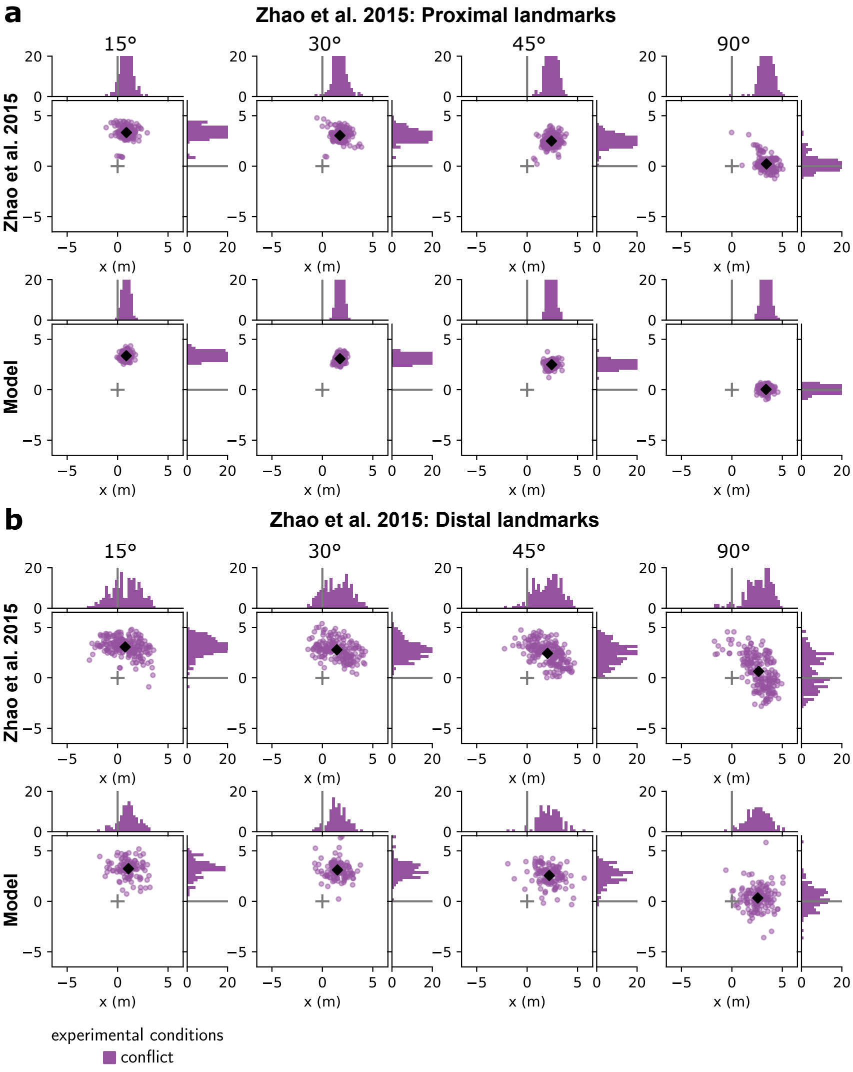

Supplementary Figure 14. Zhao et al. 2015: Conflict conditions 15-90°. a Empirical endpoint variability (top) vs. simulated endpoint variability (bottom) for varying conflict angle in the proximal landmark environment. **b** Empirical endpoint variability (top) vs. simulated endpoint variability (bottom) for varying conflict angles in the distal landmark environment.

Supplementary References

1. Nardini, M., Jones, P., Bedford, R. & Braddick, O. Development of Cue Integration in Human Navigation. en. *Current Biology* **18,** 689–693. ISSN: 09609822. https://linkinghub.elsevier.com/retrieve/pii/S096098220800506X (2020) (May 2008).

2. Zhao, M. & Warren, W. H. How You Get There From Here: Interaction of Visual Landmarks and Path Integration in Human Navigation. en. *Psychological Science* **26,** 915–924. ISSN: 0956-7976, 1467-9280. http://journals.sagepub.com/doi/10.1177/0956797615574952 (2020) (June 2015).

3. Chen, X., McNamara, T. P., Kelly, J. W. & Wolbers, T. Cue combination in human spatial navigation. en. *Cognitive Psychology* **95,** 105–144. ISSN: 00100285. https://linkinghub.elsevier.com/retrieve/pii/S0010028516302043 (2020) (June 2017).

4. Kallie, C. S., Schrater, P. R. & Legge, G. E. Variability in stepping direction explains the veering behavior of blind walkers. en. *Journal of Experimental Psychology: Human Perception and Performance* **33,** 183–200. ISSN: 1939-1277, 0096-1523. http://doi.apa.org/getdoi.cfm?doi=10.1037/0096-1523.33.1.183 (2021) (2007).

5. Consolo, P., Holanda, H. C. & Fukusima, S. S. Humans tend to walk in circles as directed by memorized visual locations at large distances. en. *Psychology & Neuroscience* **7,** 269–276. ISSN: 1983-3288, 1984-3054. http://doi.apa.org/getdoi.cfm?doi=10.3922/j.psns.2014.037 (2021) (2014).

6. Jürgens, R., Boss, T. & Becker, W. Estimation of self-turning in the dark: comparison between active and passive rotation. *Experimental Brain Research* **128,** 491–504 (1999).

7. Belousov, B., Neumann, G., Rothkopf, C. A. & Peters, J. R. *Catching heuristics are optimal control policies* in *Advances in Neural Information Processing Systems* (eds Lee, D., Sugiyama, M., Luxburg, U., Guyon, I. & Garnett, R.) **29** (Curran Associates, Inc., 2016). https://proceedings.neurips.cc/paper/2016/file/43fa7f58b7eac7ac872209342e62e8f1-Paper.pdf.

8. Mallot, H. A. & Lancier, S. Place recognition from distant landmarks: human performance and maximum likelihood model. en. *Biological Cybernetics* **112,** 291–303. ISSN: 0340-1200, 1432-0770. http://link.springer.com/10.1007/s00422-018-0751-4 (2021) (Aug. 2018).

9. Carlisle, R. E. & Kuo, A. D. Optimization of energy and time predicts dynamic speeds for human walking. *Elife* **12,** e81939 (2023).
