# Supplementary material for: Human navigation strategies and their errors result from dynamic interactions of spatial uncertainties
Source: Nat Commun. 2024 Jul 6;15:5677. doi: 10.1038/s41467-024-49722-y (PMC11227593; doi:10.1038/s41467-024-49722-y)
Supplement: Supplementary file 3 — Description of Additional Supplementary Files [file 41467_2024_49722_MOESM3_ESM.pdf]

Description of Additional Supplementary Files

Supplementary Movie 1:

Visualization of navigation strategies which emerge in optimal control under uncertainty depending on the availability and reliability of spatial cues. Blue ellipses show spatial uncertainties regarding position of the navigator a nd internally represented positions of landmarks and previously visited object locations. Based on int ernal representation paths to navigational goals can be planned (vector-based navigation). The belief state keeps track of position and the associated positional uncertainty taking into account changes in linear and angular velocity and their magnitude (path-integration). Walking to wards a visible goal allows for correcting noisy motor actions and reaching the navigational goal succe ssfully (beaconing). Landmarks provide orientation about position and heading direction (landmark-based navigation).

Supplementary Movie 2:

Visualization of triangle completion (human vs. model behavior) for different experimental conditions (self-motion, combined, conflict) for (simulated) participants in the proximal environment by Zhao et. al 2015.

Supplementary Movie 3:

Visualization of model behavior in the triangle completion for different experimental conditions (land mark, self-motion, combined, conflict 15°) in the proximal environment by Zhao et. al 2015.

Supplementary Movie 4:

Visualization of model behavior in the triangle completion for different conflict conditions (15°, 30°, 4 5°, 90°) in the proximal environment by Zhao et. al 2015.
