# Supplementary material for: Human navigation strategies and their errors result from dynamic interactions of spatial uncertainties
Source: Nat Commun. 2024 Jul 6;15:5677. doi: 10.1038/s41467-024-49722-y (PMC11227593; doi:10.1038/s41467-024-49722-y)
Supplement: Supplementary file 8 — Reporting Summary [file 41467_2024_49722_MOESM8_ESM.pdf]

nature portfolio

Corresponding author(s): Fabian Kessler, Constantin A. Rothkopf

Last updated by author(s): Apr 30, 2024

Reporting Summary

Nature Portfolio wishes to improve the reproducibility of the work that we publish. This form provides structure for consistency and transparency in reporting. For further information on Nature Portfolio policies, see our Editorial Policies and the Editorial Policy Checklist.

Statistics

For all statistical analyses, confirm that the following items are present in the figure legend, table legend, main text, or Methods section.

n/a	Confirmed	
☐	☒	The exact sample size (*n*) for each experimental group/condition, given as a discrete number and unit of measurement
☐	☒	A statement on whether measurements were taken from distinct samples or whether the same sample was measured repeatedly
☐	☒	The statistical test(s) used AND whether they are one- or two-sided<br>*Only common tests should be described solely by name; describe more complex techniques in the Methods section.*
☐	☒	A description of all covariates tested
☐	☒	A description of any assumptions or corrections, such as tests of normality and adjustment for multiple comparisons
☐	☒	A full description of the statistical parameters including central tendency (e.g. means) or other basic estimates (e.g. regression coefficient) AND variation (e.g. standard deviation) or associated estimates of uncertainty (e.g. confidence intervals)
☐	☒	For null hypothesis testing, the test statistic (e.g. *F*, *t*, *r*) with confidence intervals, effect sizes, degrees of freedom and *P* value noted<br>*Give P values as exact values whenever suitable.*
☒	☐	For Bayesian analysis, information on the choice of priors and Markov chain Monte Carlo settings
☒	☐	For hierarchical and complex designs, identification of the appropriate level for tests and full reporting of outcomes
☐	☒	Estimates of effect sizes (e.g. Cohen's *d*, Pearson's *r*), indicating how they were calculated

Our web collection on statistics for biologists contains articles on many of the points above.

Software and code

Policy information about availability of computer code

Data collection	Data in this research were generated based on computational models implemented in custom software written in Python and available from the software repository (https://doi.org/10.5281/zenodo.11090488)
Data analysis	Data were analyzed using custom Python code . We used the following packages for preprocessing of data: numpy (.17.2), pandas (0.25.1). Statistical tests were performed using pingouin (0.5.1) and dcor (0.6).

For manuscripts utilizing custom algorithms or software that are central to the research but not yet described in published literature, software must be made available to editors and reviewers. We strongly encourage code deposition in a community repository (e.g. GitHub). See the Nature Portfolio guidelines for submitting code & software for further information.

Data

Policy information about availability of data

All manuscripts must include a data availability statement. This statement should provide the following information, where applicable:

- Accession codes, unique identifiers, or web links for publicly available datasets
- A description of any restrictions on data availability
- For clinical datasets or third party data, please ensure that the statement adheres to our policy

Human datasets were recorded in previous studies (Nardini 2008, Zhao 2015, Chen 2017) and obtained from correspondence with the respective studies main author. To our knowledge these data have not been made available publicly as of yet.

Nardini, M., Jones, P., Bedford, R., & Braddick, O. (2008). Development of cue integration in human navigation. Current biology, 18(9), 689-693.

Zhao, M., & Warren, W. H. (2015). How you get there from here: Interaction of visual landmarks and path integration in human navigation. Psychological science, 26(6), 915-924.

Chen, X., McNamara, T. P., Kelly, J. W., & Wolbers, T. (2017). Cue combination in human spatial navigation. Cognitive Psychology, 95, 105-144.

Model simulated data for all 5 experiments and analysis script are available from our manuscripts accompanying github repository (https://doi.org/10.5281/zenodo.11090488).

Research involving human participants, their data, or biological material

Policy information about studies with human participants or human data. See also policy information about sex, gender (identity/presentation), and sexual orientation and race, ethnicity and racism.

Reporting on sex and gender	No experimental data was recorded for this manuscript. Human data obtained from previous work were not analyzed based on any of the above criteria.
Reporting on race, ethnicity, or other socially relevant groupings	No experimental data was recorded for this manuscript. Human data obtained from previous work were not analyzed based on any of the above criteria.
Population characteristics	Further details regarding population characteristics can be obtained from the original studies (Nardini 2008, Zhao 2015, Chen 2017.)
Recruitment	Details regarding recruitment can be obtained from the original studies (Nardini 2008, Zhao 2015, Chen 2017)
Ethics oversight	All three studies report approval by local ethics council in their publications (Nardini 2008, Zhao 2015, Chen 2017).

Note that full information on the approval of the study protocol must also be provided in the manuscript.

Field-specific reporting

Please select the one below that is the best fit for your research. If you are not sure, read the appropriate sections before making your selection.

☐ Life sciences ☒ Behavioural & social sciences ☐ Ecological, evolutionary & environmental sciences

For a reference copy of the document with all sections, see nature.com/documents/nr-reporting-summary-flat.pdf

Behavioural & social sciences study design

All studies must disclose on these points even when the disclosure is negative.

Study description	Quantitative Data in this research were generated based on computational models implemented in custom software written in Python. This model simulates goal-directed navigation behavior for 5 different experiments.<br><br>Human Datasets were obtained from direct correspondence with authors of previously published studies. To our knowledge these data have not been made available publicly as of yet. For details regarding the human data please refer to the original studies (Nardini 2008, Zhao 2015, Chen 2017).
Research sample	No experimental data was recorded for this manuscript. All three studies recorded subjects within a university setting. Details regarding the research sample can be obtained from the original studies (Nardini 2008, Zhao 2015, Chen 2017).
Sampling strategy	No experimental data was recorded for this manuscript. Details regarding the sampling strategy can be obtained from the original studies (Nardini 2008, Zhao 2015, Chen 2017).
Data collection	No experimental data was recorded for this manuscript Details regarding the data collection can be obtained from the original studies (Nardini 2008, Zhao 2015, Chen 2017).
Timing	No experimental data was recorded for this manuscript. Details regarding the timing of data collection can be obtained from the original studies (Nardini 2008, Zhao 2015, Chen 2017).
Data exclusions	Specific human datasets from each study (Nardini 2008, Zhao 2015, Chen 2017) were chosen for model simulations for illustration of our models expressiveness. Individual endpoints were classified as outlier removed based on 1.5 IQR criterion used in the original studies.

Non-participation	No experimental data was recorded for this manuscript. Details regarding the participant dropout can be obtained from the original studies (Nardini 2008, Zhao 2015, Chen 2017).
Randomization	No experimental data was recorded for this manuscript. Details regarding randomization procedures can be obtained from the original studies (Nardini 2008, Zhao 2015, Chen 2017).

Reporting for specific materials, systems and methods

We require information from authors about some types of materials, experimental systems and methods used in many studies. Here, indicate whether each material, system or method listed is relevant to your study. If you are not sure if a list item applies to your research, read the appropriate section before selecting a response.

Materials & experimental systems

n/a	Involved in the study
☒	☐ Antibodies
☒	☐ Eukaryotic cell lines
☒	☐ Palaeontology and archaeology
☒	☐ Animals and other organisms
☒	☐ Clinical data
☒	☐ Dual use research of concern
☒	☐ Plants

Methods

n/a	Involved in the study
☒	☐ ChIP-seq
☒	☐ Flow cytometry
☒	☐ MRI-based neuroimaging
